# Barriers to and Facilitators of Antiretroviral Therapy Adherence in Nepal: A Qualitative Study

**DOI:** 10.3329/jhpn.v30i4.13294

**Published:** 2012-12

**Authors:** Sharada P. Wasti, Padam Simkhada, Julian Randall, Jennifer V Freeman, Edwin van Teijlingen

**Affiliations:** ^1^School of Health and Related Research (ScHARR), University of Sheffield, Sheffield, UK;; ^2^Business School, University of Aberdeen, Aberdeen, UK;; ^3^Health and Social Care, Bournemouth University, Bournemouth, UK

**Keywords:** Adherence, Antiretroviral drugs, Barriers, Facilitators, HIV/AIDS, South Asia

## Abstract

Patient's adherence is crucial to get the best out of antiretroviral therapy (ART). This study explores in-depth the barriers to and facilitators of ART adherence among Nepalese patients and service providers prescribing ART. Face-to-face semi-structured interviews were conducted with 34 participants. Interviews were audio-taped, transcribed, and translated into English before being analyzed thematically. ART-prescribed patients described a range of barriers for failing to adhere to ART. Financial difficulties, access to healthcare services, frequent transport blockades, religious/ritual obstacles, stigma and discrimination, and side-effects were the most-frequently discussed barriers whereas trustworthy health workers, perceived health benefits, and family support were the most-reported facilitators. Understanding barriers and facilitators can help in the design of an appropriate and targeted intervention. Healthcare providers should address some of the practical and cultural issues around ART whilst policy-makers should develop appropriate social policy to promote adherence among ART-prescribed patients.

## INTRODUCTION

Antiretroviral therapy (ART) has dramatic positive effects on the lifespan and quality of life of people living with HIV (PLWH). ART reduces the load of human immunodeficiency virus (HIV) and increases CD4 cell counts, delaying progression to acquired immune deficiency syndrome (AIDS) and reducing morbidity and mortality (1). Since 2004, Nepal has been providing free-of-charge ART nationwide but the cost of diagnostic tests and travel costs must be paid for by patients (2). By the end of 2009, over 2,586 adults out of an estimated 63,528 PLWH received free ART at 23 sites across the country (4.1%) (3).

The distribution of HIV prevalence across the country is uneven. Almost 50% of all PLWH live in the highway districts, followed by 19% in the hill districts and 16% in the Far-western and Kathmandu valley (3). Despite numerous efforts by the Government of Nepal, the uptake of ART is poor due to a range of barriers and obstacles, including geographical barriers, limited infrastructure (i.e. lack of facilities for CD4 assessment, clinicians, and adequate space), financial constraints, and weak leadership and management at the national level (4). These barriers may also affect the adherence to ART by PLWH in Nepal.

Simply making ART available to PLWH is not enough as strict adherence is required for treatment success (5). Adherence is vital to the effectiveness of the ART as it is associated with clinically-significant reduction in viral load, lower drug resistance, and slower progression to AIDS (6,7). Unfortunately, up to 25% of patients do not adhere to ART (8). Both developed and developing countries have found a range of barriers to both starting and maintaining treatment, which relate to patients, healthcare systems, costs, stigma and discrimination, and treatment regimens (9-12). Facilitators of ART include financial assistance, good relationship with care providers, family support, and disclosure of HIV status to others (10,12).

It is important to study adherence in the cultural context of applying the theory of attribution (13) because Nepal is a multicultural and multi-ethnic society (14) where beliefs about health and illness vary between population groups. People's beliefs and social knowledge help them interpret events, derive meaning from them, and ascribe value to them within their social group (15). Culture is the collective consciousness that defines individual behaviours and leads to the interpretation of the world around us (16). It is described as social glue and is a socially-constructed phenomenon which can be shaped by groups that people belong to (17). Understanding culture as an internal (i.e. values, rules) or external (national culture) variable can help improve adherence. These two variables are keys to understanding the patterns of assumptions, beliefs, practices, and artifacts (18,19). The theory of attribution can help us understand these cultural issues and how they affect adherence to ART, how people explain their experiences and how attributions may influence their behaviour (20). It seems plausible that individuals who share similar basic assumptions interpret events in similar ways. For example, if patients adhere, they attribute this to internal strength whereas if they fail, rather than blaming themselves they attribute this to situational factors, such as being busy, financial difficulties, pills run-out, and side-effects (20,21). Thus, adherence is attributed to internal stable factors while non-adherence is attributed to external and, hence, uncontrollable factors (20). As a result, this study will focus on both the internal and external factors that promote or prevent adherence to ART. We are not aware of a similar study of this type conducted in Nepal.

## MATERIALS AND METHODS

Qualitative research is widely used in health and social sciences around sensitive issues (22), and HIV in Nepal is stigmatized and sensitive (4). We examined the views of PLWH in their natural settings to identify contextual influences of adherence to ART (23,24). Purposive sampling technique was used in selecting 34 interviewees for face-to-face semi-structured interviews. This method is often used when the researcher has specific requirements for the sample and picks a subject who meets these strict criteria (24). The sample comprised 17 ART-prescribed patients, 14 ART service providers (doctors, nurses, and paramedics), and three policy-makers. ART prescribed patients were recruited through accessing medical records for patients who missed refill prescription as well as those who never missed. Similarly, ART service providers and key policy-makers were identified, who were closely working with the ART programme. Data were considered saturated when no more new information could be obtained (25).

Interviews were conducted with the aid of an interview checklist and included probes for further questioning (24), covering questions on how ART fitted into their daily routines and what the key barriers to and facilitators of adherence to ART were. The interviews were conducted and audio-taped in Nepali by the first author who is a native speaker; sessions lasted one to one and-a-half hours. Audio-recordings were transcribed verbatim in Nepali and then translated into English. To ensure accuracy, the translations were cross-checked for inter-rater reliability by the second author who is also a native Nepali speaker (26). Data were organized using NVivo 8 and coded using a thematic analysis (22). The quotations cited in this paper best represented the range of ideas voiced around key themes. To maintain anonymity, these quotes are identified by gender and region alone, with a serial number designated by ‘P’. In addition, nurses and paramedics are all coded as ‘counsellor’. To assure quality of the data, the checklist was piloted with two patients on ART and a care provider. Subsequently, some modifications were incorporated in the research instruments (27). Ethical permission for the study was obtained from the Nepal Health Research Council.

## RESULTS

Major barriers of ART adherence themes were grouped into five interwoven categories: (i) economic, (ii) individual patient-related, (iii) sociocultural, (iv) healthcare provision and system, and (v) drug-related barriers.

### 1. Economic barriers

Money emerged as the greatest barrier as most respondents reported economic worries relating to the costs of transport, prescription, and charges for diagnosis. Interviewees often did not have the money to go to health facilities for repeat prescriptions (‘refill’), and, thus, transport costs emerged as a key theme. As subsistence farming was the main source of income for most PLWH, few had spare cash. One interviewee reported:

The reason… I missed my medication was.…the money. I need to manage 500 rupees (~£4) for bus fare every month to refill prescription of ART. Who gives me this amount? No money! No source of income! How can I afford this [cost]? I do not have any alternative, except stopping treatment (P-12, Female, Far-western)

Healthcare providers also concurred with this. Individuals often could not pay for travel to the ART centre before their drugs ran out. Other financial constraints were the cost of registration and laboratory tests. A patient stated:

I need to refill my ART every end of a month. How could I get a registration charge?.…every month I need to pay registration fee of 40 rupees (~50 pence) and every three months for CD4 test (~£1).…how can I get this amount? This is a big problem for me! (P-4, Female, Kathmandu)

One ART counsellor also noted that patients needed to bear the registration and diagnostic (laboratory) costs:

Patients themselves need to pay Rs. 40 (~0.40 pence) as registration charge and Rs. 100 (~£1) for CD4 test and related travel cost; some are getting support from NGOs but hospital does not have a policy to provide free diagnostic test (P-26, Counsellor, Kathmandu)

This may remain a major reason why some individuals do not come to the health facility to refill their prescriptions, even when the treatment itself is free of charge.

### 2. Individual patient-related barriers

In-depth interviews with all the participants recognized the following patient-related barriers to adherence.

### Perception about ART

Perceived self-efficacy is concerned with people's belief that they can exert control over their own motivation, through processes, emotional states, and patterns of behaviour. Some PLWH questioned the efficacy of ART. For example, one participant stated:

Rural people still do not believe this medicine [ART] works for HIV patients. HIV people will die eventually either taking or not taking ART. Why should I die by taking these malicious pills? They stopped taking medicine after initiating treatment (P-12, Female, Far-western)

Similarly, the reaction of family members affected adherence. Some interviewees stated that HIV is not currently curable and that treating PLWH was just a wastage of money. For example, one respondent said:

Some family members are not ready to get repeat prescription of ART because of HIV. There is no medicine to cure HIV. So, why should we send her to hospital? She will die soon. Why should we waste money and time? (P-14, Female, Highway)

### Substance-misuse

Misuse of substance was mentioned by PLWH as contributing to non-adherence. A female participant stated:

Some of male clients are not continuing their medication.…they are not ready to go to hospital to refill prescription. They drink alcohol from early morning. How could they remember their ART time? (P-12, Female, Far-western)

Some stated that, at festival times, they were expected to drink alcohol and that this caused difficulty with remembering their medication routine. A male disclosed:

I need to attend parties and drink during festival times.…when I drink alcohol, I have great difficult to take my night dose [of ART]. I missed it many times (P-13, Male, Hill)

Healthcare providers also agreed that some patients had reported missing ART due to alcohol and drug consumption. Substance-users did not take medications on time because their substance-misuse made them relapse or forget the timing of their medication. One counsellor reiterated this issue, thus:

It is.…a problem with a few drug-using patients because their main focus was drug. Due to the addiction, they do not care about HIV.…Drug is the most important thing for them (P-20, Counsellor, Highway)

### Simply forgetting and being busy

Patients sometimes forgot their medication due to being busy or away from home. Some forgot when they were busy with their daily routine. One male disclosed:

I missed some doses [of ART] because I simply forgot. (P-10, Male, Hill)

Similarly, a female patient mentioned being busy and away from home:

My children are very young, and last year my husband died.…I am responsible for all work. I am always very busy.…I did not reach hospital on time to collect medicine, do not have time to eat, and had missed some doses in different times (P-12, Female, Far-western)

Having responsibilities of family work, particularly for single parents, could well account for non-adherence for those with a difficult journey to the nearest hospital as these, together with their work responsibilities, impeded the adherence of some PLWH.

### 3. Sociocultural barriers

HIV is still a sensitive social and cultural issue in Nepal, and our findings supported this.

### Barriers relating to religion and rituals

People live in a community and need to abide by their local and traditional religious and cultural rituals, which can influence adherence to ART. A Muslim reported:

I stopped my morning dose of ART during Ramadan.…I was sick and went to consult the doctor, and he told me not to stop at anytime.…now I am taking medicine when fasting (P-16, Female, Highway)

Similarly, a Hindu female ART-recipient also added:

During Teej [Hindu women's fasting day], we won't drink water the whole day.…How can we take ART?.…I am fasting but I take ART in empty stomach.…What can we do? (P-5, Female, Kathmandu)

A doctor stated:

I am not blaming all my patients but a few of Hindu women during Teej and Muslim patients in Ramadan have problems taking medicine.…a few Muslim patients did not take medicine in the morning because of Ramadan. They told me if they drink water during the day, this is totally sinful (P-27, Doctor, Kathmandu)

### Lack of family support

Lack of family support acted as a barrier to adherence, and family arguments stopped them from taking medication, as one female participant explained:

I skipped two doses because of a family quarrel.…my family did not allow me to visit hospital (P-14, Female, Highway)

Some professionals also noted that patients without family support had lower adherence levels. A counsellor stated:

One patient skipped medicine because no one brought medicine for her from the hospital. She was bed-ridden but family members did not bring medicine.…Because the pills ran out and no one helped her, she skipped some doses [of ART] (P-18, Counsellor, Far-western)

Thus, the combination of difficulty with accessing the hospital and an unhelpful family can negatively affect adherence.

### Stigma and discrimination

Most participants mentioned that they had experienced some form of discrimination and HIV-related stigma (or fear of stigma), which influenced adherence; this was especially so among women:

I have not taken my medication two times because relatives and neighbours were in my house. I did not get time to take out medicine from my drawer.…I have frequent problems to hide my medicine from others because I am living in a rented single room.…Oh! I cannot tell anyone (P-5, Female, Kathmandu)

The fear of being victimized and/or rejected by their family or community generated a fear of exposure, which in itself affected adherence:

I am worried about meeting my neighbours in hospital for refills [ART]. All the time I worry: “How can I hide from these people?” One day I did not refill my ART due to bumping into relatives (P-4, Female, Kathmandu)

Some healthcare providers also noted that PLWH selected ART sites where no one would know them:

Many patients still prefer visiting the distant hospital; they are not ready to go nearby. This is all about the fear of [disclosure to] other people (P-20, Counsellor, Kathmandu)

A doctor added:

Clients are still stigmatized.…they fear other people.…‘What do they think?’, ‘What do they say?’.…clients are not ready to take medicine in front of other people.…they choose the alternative and skip medication (P-27, Doctor, Kathmandu)

### 4. Healthcare provision and system

### Distance

Nepal's large rural hinterland, combined with a limited number of ART sites, was perceived to have a negative effect on adherence. Distance was a big concern, particularly outside Kathmandu. The travelling distance to and from ART sites remained one of the most-challenging adherence issues, and it was frequently discussed by both PLWH and care providers:

I need to walk more than one and half days to refill my medicine [ART].…this is very difficult at my age, not once a year, but every month (P-1, Male, Highway)

Similarly, policy-makers agreed that distant and centralized ART-providing institutions limited adherence to ART:

Treatment services are located in a limited number of central hospitals, which is a major problem for PLWH seeking services. That's definitely limiting their adherence.…HIV treatment service is still not reaching the people in need (P-33, Policy-maker)

### Short period of medicine prescription

Having very few ART institutions is itself a barrier to getting repeat prescriptions on time. The different prescribing policies in different hospitals are also additional factors. Some clinicians prescribe ART drugs for one month, and others up to a maximum of two months. An interviewee stated:

Doctors prescribed ART only for a month. This is a very short time.…refilling ART every month is extremely difficult to adhere to this medication (P-5, Female, Kathmandu)

However, rural clinicians mostly prescribed two months of ART at a time, for example:

I have to visit this hospital every two months to refill medicine.…I have to arrange money and need to walk long distance, which is very hard not only for me but most of the PLWH [from hill districts].…to refill ART every two months is the main problem for me (P-10, Male, Hill)

### Strikes (transportation blockades)

Nepal's political situation was very volatile at the time of the study, which led to frequent and unpredictable strikes. This often meant that main roads were blocked, leading to reduced ART adherence:

Unexpected strikes are in fashion at the moment.…bus-strikes.…and last time even the hospital was on strike for a week.…How can we take regular medicine?.…Think about Nepal's geography and distance, how can all ART-recipients receive medicine without missing any?’ (P-5, Female, Kathmandu)

Service providers also agreed that, due to frequent and unexpected roadblocks, PLWH have no alternative other than to stop medication or miss some doses of their medicine.

### 5. Drug-related barriers

Side-effects of ART are one of the most-discussed themes in this study. Most participants had experienced side-effects, which increased non adherence:

ART caused a problem rather than good health. I did not tolerate the pain, and I stopped for a while (P-9, Male, Highway).

Similarly, a woman found that ART caused physical problems:

.…ART caused me severe vomiting and griddiness. I was vomiting like I was going to die. If this condition would continue, how can I adhere to ART (P-17, Female, Kathmandu)

Doctors agreed that side-effects, such as vomiting, body-pain, and skin rashes, led some patients to stop taking ART.

### Facilitators of adherence

Despite many barriers, five major themes were identified as facilitators of adherence to ART as follows: (i) trusting health workers, (ii) positive beliefs about ART, (iii) ART as a part of daily life, (iv) responsibilities for children, and (v) family and mechanical support.

### Trusting health workers

Patients’ trust in doctors and their guidance motivated them to continue their medication. Supportive and interested practitioners reinforced good practice in their patients. Some PLWH felt respect for and support from healthcare staff. Where patients felt that their doctors had faith in them, their own hopes increased, and they believed more in their treatment. A male participant stated:

The doctor counsels me so nicely. He told me HIV-positives are living longer, nobody will die soon due to HIV. A disease can happen to anyone, either rich or poor. ART will make you healthier and live longer. He is very worried about my medication. I could not stop it any time (P-13, Male, Highway)

One doctor spoke of patients having a higher trust in the medical profession than other counsellors:

They [PLWH] have good trust in doctors. They follow the doctors’ suggestions more strictly compared to other health workers (P-27, Doctor, Kathmandu)

### Positive beliefs about ART

Fear of doing worse without adhering to ART was a prime motivator for adherence. Many patients were very optimistic about ART efficacy:

I do not want to imagine my life without this [ART] (P-3, Male, Kathmandu)

Another participant highlighted that PLWH do not have a choice:

I have two choices—whether I want to live or die. If I want to live longer, I must need to take ART, which I am doing and will be doing until another medicine (P-11, Male, Far-western)

Participants reported ART had increased their body-weight and appetite, reduced opportunistic infections, and improved their quality of life.

### ART as a part of daily life

Participants who could link ART to their daily routines found it much easier to remember to take it:

I carry ART anywhere because I may go out and sometimes be late coming home. I must take these on time. See my bag, I have ART now (P-3, Male, Kathmandu)

Many had found strategies to incorporate taking medication into their lifestyle:

Oh! ART is my life and is a part of my life just like the morning teeth brushing. My inner sight pushes me always that I have to take my medicine on time to feel good (P-14, Female, Highway)

### Responsibilities for children

Caring for a family and having responsibility for looking after children was one of the most frequently-cited facilitators of adherence:

I have the responsibility to care for my children. I need to get my daughter married. She is 16 years old now (P-14, Female, Highway)

Healthcare providers also expressed the opinion that PLWH who worried about the future of their children showed a relatively good adherence:

The motivation of patients for taking ART is usually to be healthy and live longer because they want to and care for their families (P-22, Counsellor, Highway)

### Family and mechanical support

Better understanding among family members was spoken of as something that improved adherence. An interviewee recalled how her family helped her:

.…I often forgot to take ART, my daughter asked me, mummy, have you taken your medicine today?.… She reminds me. Almost all the time she [daughter] brings a glass of water and pack of medicines (P-12, Female, Far-western)

Families also provide financial support:

I do not need to worry about any ART-related costs. My parents give me money and support everything.…I am alive today; it is because of my family (P-15, Female, Hill)

Some participants used electronic devices to remind them to take their daily medication and found this support useful:

I set different alarm tones in my mobile.…I have recorded my own voice; It is time (*Samaya bhayo*), this alerts me (P-7, Male, Kathmandu)

## DISCUSSION

### Barriers

Some of the barriers identified in this study are consistent with the literature from both developed and developing countries (10,12). Although ART drugs have been provided free of charge in Nepal, ART-associated costs (including transport, prescription, and diagnostic tests) were by far the most-discussed theme relating to non-adherence as has been found elsewhere (12,28,29). Large income disparities persist across ecological zones, rural and urban locations, and by caste and ethnicity, which affect all aspects of care, including patients’ ability to meet the ART-associated costs. One study from Africa has suggested that poor patients are able to achieve excellent rates of adherence with free laboratory monitoring and subsidized ART (30). This is a useful finding, and it is important that policy-makers in Nepal should consider subsidies for transport and other costs associated with ART when designing treatment programmes. Our interviewees were concerned that physicians often prescribed ART for periods that were too short, thus increasing costs associated with transportation to collect ART drugs. Again, this is a finding that has been documented elsewhere (6). Patients were worried and reported that refilling ART prescription every month caused difficulties in adherence. The Government of Nepal needs to consider carefully the amount of medicine prescribed on each occasion as well as subsidizing the travel costs. Making available ART drug-collection points in district hospitals rather than a limited numbers of selected central-level hospitals may also help alleviate the (travel) cost issues around having only a short period between refills.

Most interviewees stated that, although patients were willing to take ART, they became non-adherent because of difficulties in reaching the treatment centres due to unexpected transport strikes; long travel distance; geographical difficulty, including lack of transportation services in many remote areas; and the seasonal deterioration of poorer road conditions during the rainy season. This has also been found to be the case with respect to maternity services in Nepal (31). Various studies have also found that travel time and access to treatment centres were barriers to adherence elsewhere (32,33) and that better access to care was significantly associated with optimal adherence (34). Interestingly, our findings revealed that, in urban areas, the problem was related to transportation blocks, while in rural areas, the difficulties were more about the long travel distance and the lack of availability of transportation services. The current HIV/AIDS treatment programme in Nepal has been running in a limited number of ART sites and has largely ignored the existing public-health networks within the community. Therefore, any new policy needs to overcome the reduced access to medical care services by integrating ART into the mainstream healthcare rather than concentrating treatment in a limited number of ART centres, which may be hard to reach for the majority of patients.

Patients’ beliefs, knowledge, and expectations regarding treatment strongly influence their medical decision-making (35). Our findings show that some participants questioned the efficacy of ART. PLWH who believed in the efficacy of ART are more likely to adhere (36), and both education level of PLWH and educational programmes affect adherence (37). Providing better information about HIV/AIDS reduces both fear and ignorance so that patients maintain their medication better (38). Our study revealed that alcohol and illicit drug-use increased non-adherence, and similar findings have been reported elsewhere (12,36,39). In addition, one study showed that not using substances increased adherence (40).

Other barriers to adherence raised during the interviews included local cultural factors, especially religious activities and festivals, such as Teej for Hindu women and Ramadan for Muslims. One possible intervention could come from Hindu and Muslim clergy who could stress the importance of continuing to take medicine during religious festivals. Our findings reinforce the importance of considering the religious and spiritual beliefs of PLWH as part of medical care. It is believed that most religions give freedom to eat on fasting days, especially for the sick, children, and older people. Hence, this message needs to be reinforced during counselling.

Interviewees reported that stigma and discrimination was still widespread from both their own family and their local community. Some participants did not take medicine in front of people they knew due to fear of being identified as HIV-positive (41). Elsewhere, PLWH were unwilling to seek treatment at the nearest health facility because of fear of stigmatization (42). In addition, the threat of social stigma may prevent PLWH from revealing their status to others (43). It has been shown that PLWH who do not disclose their status to their family members or peers did worse in their treatment than those who disclosed (10). It is an important aspect of the development of evidence-based interventions targeted at non-adhering individuals that they are encouraged to disclose as this is linked with positive adherence practice, a result that has also been seen elsewhere (44).

ART regimens have toxicities and adverse side-effects that vary from mild to severe and acute to chronic, which can prevent adherence (45). In our study, patients who had side-effects were more likely to be non-adherent, and this has also been reported in several other studies conducted in both developed and developing countries (10,12). Hence, there is a need for continuing follow-up, clinical support, and counselling for patients who just started ART and for follow-up appointments to include instructions on how to cope with side-effects (46). Practitioners should advise all clients on how to handle side-effects so that these cannot adversely influence ART adherence.

### Facilitating factors

Some of our interviewees were motivated to continue their ART by a desire to live longer and remain healthy for longer period. Tangible and emotional support delivered in culture-specific ways can be of help to foster adherence (47), for example, improving internal affective states, such as beliefs and perceptions of positive results of ART (48). Healthcare workers should continuously offer information and counselling to (a) prevent patients from stopping treatment and (b) make intake of medication a daily priority (46).

Participants discussed the two-fold nature of social support systems as a barrier and as a facilitator of adherence. Some people were accepting and supportive while others overtly or subtly distanced themselves from PLWH. Our findings highlighted that family support increased the likelihood of patients to maintain optimal adherence. In addition, social and family support and access to care that anticipate patients’ individual needs play an important positive role (34,49). Particularly for women and children, family acted as a facilitator for adherence. A meta-analysis reported that adherence is 1.74 times higher in patients from cohesive families (50). Some of our interviewees used electronic aids (alarm, mobile phone) as a tool to remind them of their medication, and others have shown that the use of such aids improved adherence (51). Therefore, healthcare providers should discuss with new starters and individuals in follow-up about the use of these aids.

Finally, healthcare workers played an important role in supporting and encouraging patients to adhere to their medication. Good relationships with care providers and trust in them improved adherence as has been found elsewhere (46). Healthcare providers who spent time explaining things to patients positively influence adherence and, thus, it is possible that time spent talking to significant influencing groups would help reinforce adherence (52). Service providers should promote optimal adherence by giving clear instructions, providing adequate medical follow-ups that address possible side-effects and how to handle these in order to reinforce adherence. Supportive and interested practitioners can motivate and reinforce good practice in their patients.

### Conclusions

Successful adherence to ART is highly dependent on patients’ beliefs about the treatment, and subsequent treatment-seeking behaviours in practice. Our study revealed a range of barriers to adherence: financial difficulties, treatment access, frequent transport blockades, religious or ritual obstacles, stigma and discrimination, and side-effects. Perceived benefits and trustworthy health workers were the most-frequently reported facilitators. It is necessary to recognize and overcome the key barriers and promote measures to facilitate adherence. Priority should be given to improving adherence by providing (i) financial incentives, (ii) better access to treatment services, (iii) education and counselling to deal with religious and ritual obstacles, social stigma, and discrimination. All these are expected to be useful in overcoming the barriers to ART adherence. In addition, healthcare providers should explain the side-effects and how to handle these. It is crucial to prioritize adherence because of its impact on treatment efficacy and ultimately on patients’ quality of life and life-expectancy. Policy-makers should be aware of these key barriers and consider social policy which encourages patients to achieve optimal adherence.

## ACKNOWLEDGEMENTS

We are grateful to the individuals living with HIV, who participated in this study. Our appreciation is also due to all staff working at the ART sites involved in the programme.

## References

[1] Egger M, May M, Chêne G, Phillips AN, Ledergerber B, Dabis F (2002). ART Cohort Collaboration. Prognosis of HIV-1-infected patients starting highly active antiretroviral therapy: a collaborative analysis of prospective studies. Lancet.

[2] Wasti SP, Simkhada P, Teijlingen ER (2009). Antiretroviral treatment programmes in Nepal: problems and barriers. Kathmandu Univ Med J (KUMJ).

[3] United Nations General Assembly Special Session (2010). UNGASS country progress report: Nepal.

[4] Wasti SP, Simkhada P, Randall J, van Teijlingen E (2009). Issues and challenges of HIV/AIDS prevention and treatment programme in Nepal. Global J Health Sci.

[5] Konkle-Parker DJ, Erlen JA, Dubbert PM (2008). Barriers and facilitators to medication adherence in a southern minority population with HIV disease. J Assoc Nurses AIDS Care.

[6] Tuller DM, Bangsberg DR, Senkungu J, Ware NC, Emenyonu N, Weiser SD (2010). Transportation costs impede sustained adherence and access to HAART in a clinic population in southwestern Uganda: a qualitative study. AIDS Behav.

[7] Paterson DL, Swindells S, Mohr J, Brester M, Vergis EN, Squier C (2000). Adherence to protease inhibitor therapy and outcomes in patients with HIV infection. Ann Intern Med.

[8] DiMatteo MR (2004). Variations in patients’ adherence to medical recommendations: a quantitative review of 50 years of research. Med Care.

[9] Wang X, Wu Z (2007). Factors associated with adherence to antiretroviral therapy among HIV/AIDS patients in rural China. AIDS.

[10] Mills EJ, Nachega JB, Bangsberg DR, Singh S, Rachlis B, Wu P (2006). Adherence to HAART: a systematic review of developed and developing nation patient-reported barriers and facilitators. PLoS Med.

[11] Hardon A, Davey S, Gerrits T, Hodgkin C, Irunde H, Kgatlwane J (2006). From access to adherence: the challenges of antiretroviral treatment—studies from Botswana, Tanzania and Uganda 2006.

[12] Wasti SP, van Teijlingen E, Simkhada P, Randall J, Baxter S, Kirkpatrick P (2012). Factors influencing adherence to antiretroviral treatment in Asian developing countries: a systematic review. Trop Med Int Health.

[13] Weiner B (1979). A theory of motivation for some classroom experiences. J Educ Psychol.

[14] Dahal DR (2003). Social composition of the population: caste/ethnicity and religion in Nepal. Population monograph of Nepal 2003. Vol. 1. Chapter 3.

[15] Schein EH (1985). Defining organizational culture. Classics Organ Theor.

[16] Geertz C (1973). The interpretation of cultures: selected essays.

[17] Randall J (2004). Managing change/changing managers.

[18] Meek VL (1988). Organizational culture: origins and weaknesses. Organ Stud.

[19] Hofstede G (2003). Culture's consequences: comparing values, behaviors, institutions, and organizations across nations. 2^nd^ ed..

[20] Weiner B (1985). An attributional theory of achievement motivation and emotion. Psychol Rev.

[21] Wasti SP, Randall J, Simkhada P, van Teijlingen E (2011). In what way do Nepalese cultural factors affect adherence to antiretroviral treatment in Nepal?. Health Sci J.

[22] Silverman D (2006). Interpreting qualitative data: methods for analyzing talk, text and interaction. 3^rd^ ed.

[23] Johnson RB, Onwuegbuzie AJ (2004). Mixed methods research: a research paradigm whose time has come. Educ Res.

[24] Bowling A (2002). Research methods in health: investigating health and health services. 2^nd^ ed.

[25] Glaser BG, Strauss AL (1967). The discovery of grounded theory: strategies for qualitative research.

[26] Pitchforth E, van Teijlingen E (2005). International public health research involving interpreters: a case study from Bangladesh. BMC Public Health.

[27] Van Teijlingen ER, Rennie AM, Hundley V, Graham W (2001). The importance of conducting and reporting pilot studies: the example of the Scottish Births Survey. J Adv Nurs.

[28] Nachega JB, Knowlton AR, Deluca A, Schoeman JH, Watkinson L, Efron A (2006). Treatment supporter to improve adherence to antiretroviral therapy in HIV-infected South African adults: a qualitative study. J Acquir Immune Defic Syndr.

[29] Hardon AP, Akurut D, Comoro C, Ekezie C, Irunde HF, Gerrits T (2007). Hunger, waiting time and transport costs: time to confront challenges to ART adherence in Africa. AIDS Care.

[30] Weiser S, Wolfe W, Bangsberg D, Thior I, Gilbert P, Makhema J (2003). Barriers to antiretroviral adherence for patients living with HIV infection and AIDS in Botswana. J Acquir Immune Defic Syndr.

[31] Thomas D, Messerschmidt L, Messerschmidt D, Devkota B (2004). Increasing access to essential obstetric care: a review of progress and process.

[32] Posse M, Meheus F, van Asten H, van der Ven A, Baltussen R (2008). Barriers to access to antiretroviral treatment in developing countries: a review. Trop Med Int Health.

[33] Alker AP, Delvaux T, Mbuyi N, Ryder RW (2004). Barriers to HIV treatment of women in Kinshasa, DRC. Paper presented on XV International AIDS Conference, Bangkok, 11-16 July.

[34] Li L, Lee SJ, Wen Y, Lin C, Wan D, Jiraphongsa C (2010). Antiretroviral therapy adherence among patients living with HIV/AIDS in Thailand. Nurs Health Sci.

[35] Rabkin JG, Chesney MA (1998). Adhering to complex regimens for HIV. GMHC Treat Issues.

[36] Murphy DA, Roberts KJ, Hoffman D, Molina A, Lu MC (2003). Barriers and successful strategies to antiretroviral adherence among HIV-infected monolingual Spanish-speaking patients. AIDS Care.

[37] Kleeberger CA, Phair JP, Strathdee SA, Detels R, Kingsley L, Jacobson LP (2001). Determinants of heterogeneous adherence to HIV-antiretroviral therapies in the multicenter AIDS cohort study. J Acquir Immune Defic Syndr.

[38] Obermeyer CM, Osborn M (2007). The utilization of testing and counseling for HIV: a review of the social and behavioral evidence. Am J Public Health.

[39] Hendershot CS, Stoner SA, Pantalone DW, Simoni JM (2009). Alcohol use and antiretroviral adherence: review and meta-analysis. J Acquir Immune Defic Syndr.

[40] Lucas GM, Gebo KA, Chaisson RE, Moore RD (2002). Longitudinal assessment of the effects of drug and alcohol abuse on HIV-1 treatment outcomes in an urban clinic. AIDS.

[41] Rao D, Kekwaletswe TC, Hosek S, Martinez J, Rodriguez F (2007). Stigma and social barriers to medication adherence with urban youth living with HIV. AIDS Care.

[42] Adeneye AK, Adewole TA, Musa AZ, Onwujekwe D, Odunukwe NN, Araoyinbo ID (2006). Limitations to access and use of antiretroviral therapy (ART) among HIV positive persons in Lagos, Nigeria. World Health Popul.

[43] Rintamaki LS, Davis TC, Skripkauskas S, Bennett CL, Wolf MS (2006). Social stigma concerns and HIV medication adherence. AIDS Patient Care STDS.

[44] Kaleeba N, Kalipeni E, Craddock S, Oppong JR, Ghosh J (2004). Excerpt from we miss you all: AIDS in the family. HIV and AIDS in Africa: beyond epidemiology.

[45] Catz SL, Kelly JA, Bogart LM, Benotsch EG, McAuliffe TL (2000). Patterns, correlates, and barriers to medication adherence among persons prescribed new treatments for HIV disease. Health Psychol.

[46] Lewis MP, Colbert A, Erlen J, Meyers M (2006). A qualitative study of persons who are 100% adherent to antiretroviral therapy. AIDS Care.

[47] Ka'opua L (2001). Treatment adherence to an antiretroviral regime: the lived experience of Native Hawaiians and kokua. Pac Health Dialog.

[48] Remien RH, Hirky AE, Johnson MO, Weinhardt LS, Whittier D, Le GM (2003). Adherence to medication treatment: a qualitative study of facilitators and barriers among a diverse sample of HIV+ men and women in four US cities. AIDS Behav.

[49] Kumarasamy N, Safren SA, Raminani SR, Pickard R, James R, Krishnan AK (2005). Barriers and facilitators to antiretroviral medication adherence among patients with HIV in Chennai, India: a qualitative study. AIDS Patient Care STDS.

[50] DiMatteo MR (2004). Social support and patient adherence to medical treatment: a meta-analysis. Health Psychol.

[51] Golin CE, Liu H, Hays RD, Miller LG, Beck CK, Ickovics J (2002). A prospective study of predictors of adherence to combination antiretroviral medication. J Gen Intern Med.

[52] Coetzee D, Boulle A, Hildebrand K, Asselman V, Van Cutsem G, Goemaere E (2004). Promoting adherence to antiretroviral therapy: the experience from a primary care setting in Khayelitsha, South Africa. AIDS.

